# The County Infirmaries and the College

**Published:** 1917-10-13

**Authors:** 


### NURSING AFFAIRS IN IRELAND.

### THE COUNTY INFIRMARIES AND THE COLLEGE.

It appears that the resolutions of the Irish county infirmary surgeons with regard to the training and registration of nurses published in the Dublin press were inaccurate and unauthorised. The following is the official text :

"1. That there should be a separate Irish Board to control the curriculum of training and the registration of Irish nurses.

"2. That nurses should be educated and trained under a certain curriculum, and when their training is completed, be examined by a Board set up for that purpose in suitable centres.

"3. That any Irish hospital approved by the Local Government Board as a suitable training-school for nurses should be recognised.

"4. We consider any well-equipped Irish county infirmary affords every facility necessary for training nurses.

"5. We recommend our committees of management to oppose, and request the County Members of Parliament to oppose, any Bill which would exclude the Irish county infirmaries from training nurses."

It would appear to me that the Irish Board of the College of Nursing should take this matter up with vigour, and with a view to co-operation. Obviously there is not the smallest necessity for opposition or for friction. Last week I set out the bed accommodation of these institutions, the majority of which (for the period of grace) come within the requirements of the College, which are as follows :

"For the purpose of admitting practising nurses to the Register of the College, general civil hospitals and infirmaries are recognised for training in which

"(*a*) There is an average number of not fewer than forty beds in daily occupation throughout the year.

"(*b*) There is a resident medical or surgical officer.

"(*c*) There is given at least one course of nursing lectures annually, followed by examination for qualification."

### No Attempt at Exclusion.

These county infirmaries must not be confounded with Poor-Law infirmaries, which are on quite a different basis, and for which a minimum of 250 beds is necessary. As a matter of fact, the College of Nursing, during this period of grace, has already recognised at least ten of these county infirmaries by admitting to the Register nurses trained there. These include the County and City of Waterford, Downpatrick, Antrim, Limerick, Londonderry, Clare, Mayo, Tyrone, Donegal, and Fermanagh county infirmaries. Galway, which may be described as a university town, falls below the standard of the College, inasmuch as the necessary course of lectures is not delivered. This clearly demonstrates that the College of Nursing Bill will not attempt to exclude the Irish county infirmaries, but merely such of them as fail to come up to the minimum standard set forth above.

### The County Surgeons' Views Elaborated.

An interesting letter from Mr. Edward Thompson, F.R.C.S.I., of Omagh, Co. Tyrone, was published last week in the *Irish Times*, in which he elaborates the county surgeons' views. He says that he makes no claim that the nurses trained in their institutions are white swans, and that their imperfections are quite as apparent as those of the nurses trained in other institutions. "Our object," he writes, "is plain and above-board and should receive the support of all independent men and women, and especially of nurses trained and contemplating training. We Irish surgeons have no personal axe to grind, and have adopted our present attitude to safeguard the interest both of the State and the nursing profession, and the institutions connected with the training of nurses. We desire the State, in consultation with those best qualified to give advice, to formulate a definite scheme for the education of nurses and for their subsequent registration. There is surely nothing very alarming in this proposition. It has been already carried out in dealing with the medical profession. . . . Surely it would establish uniformity of qualification, ensure fair dealing towards the nurses, and make 'bossism' and the traffic in nurses impossible. . . . The true way to solve all these troublesome nursing questions is to institute a uniform State system of training and examination, and subsequently State registration, and that these duties should not be entrusted to any colleges, boards, committees, or private individuals, but should each and all be controlled in the first instance by the State."

### A General Awakening.

Mr. Thompson's arguments are perfectly fair, but it must not be forgotten that they are based altogether on the spade-work partly accomplished and still being continued by the College of Nursing. Public action invariably precedes State action, and it is clear to everybody in Ireland that but for the establishment here of the Irish Board of the College the whole nursing problem would be still quiescent. Hence these questions that are arising can only be regarded as symptoms of a general awakening. If the infirmaries are wise they will begin to grade up, so that when the movement is ripe, the inspecting authorities will not have to repeat their verdict of "some middling, some bad, and some very bad."

### Public Meeting in Dublin.

A golden opportunity for all concerned to come to a common understanding will shortly occur, for on October 24 a public meeting will be held in Dublin for the discussion of the aims of the College and the outlook of State Registration. Medical men and others interested in the question should attend and endeavour to establish a basis of agreement. I am very glad to learn, moreover, that at this meeting nurses will be invited to discuss the economic side of their career. It is hoped that they will find a champion for their cause. If it is left to the casual individual who may happen to be present, no better progress is likely to ensue, as very few nurses of the rank and file will find courage to address a meeting where a considerable number of doctors and matrons are assembled.

IERNE.

